# Differential gene expression in incompatible interaction between wheat and stripe rust fungus revealed by cDNA-AFLP and comparison to compatible interaction

**DOI:** 10.1186/1471-2229-10-9

**Published:** 2010-01-12

**Authors:** Xiaojie Wang, Wei Liu, Xianming Chen, Chunlei Tang, Yanling Dong, Jinbiao Ma, Xueling Huang, Guorong Wei, Qingmei Han, Lili Huang, Zhensheng Kang

**Affiliations:** 1 College of Plant Protection and Shaanxi Key Laboratory of Molecular Biology for Agriculture, Northwest A&F University, Yangling, Shaanxi, 712100, PR China; 2 USDA-ARS and Department of Plant Pathology, Washington State University, Pullman, WA 99164-6430, USA

## Abstract

**Background:**

Stripe rust of wheat, caused by *Puccinia striiformis *f. sp. *tritici *(*Pst*), is one of the most important diseases of wheat worldwide. Due to special features of hexaploid wheat with large and complex genome and difficulties for transformation, and of *Pst *without sexual reproduction and hard to culture on media, the use of most genetic and molecular techniques in studying genes involved in the wheat-*Pst *interactions has been largely limited. The objective of this study was to identify transcriptionally regulated genes during an incompatible interaction between wheat and *Pst *using cDNA-AFLP technique

**Results:**

A total of 52,992 transcript derived fragments (TDFs) were generated with 64 primer pairs and 2,437 (4.6%) of them displayed altered expression patterns after inoculation with 1,787 up-regulated and 650 down-regulated. We obtained reliable sequences (>100 bp) for 255 selected TDFs, of which 113 (44.3%) had putative functions identified. A large group (17.6%) of these genes shared high homology with genes involved in metabolism and photosynthesis; 13.8% to genes with functions related to disease defense and signal transduction; and those in the remaining groups (12.9%) to genes involved in transcription, transport processes, protein metabolism, and cell structure, respectively. Through comparing TDFs identified in the present study for incompatible interaction and those identified in the previous study for compatible interactions, 161 TDFs were shared by both interactions, 94 were expressed specifically in the incompatible interaction, of which the specificity of 43 selected transcripts were determined using quantitative real-time polymerase chain reaction (qRT-PCR). Based on the analyses of homology to genes known to play a role in defense, signal transduction and protein metabolism, 20 TDFs were chosen and their expression patterns revealed by the cDNA-AFLP technique were confirmed using the qRT-PCR analysis.

**Conclusion:**

We uncovered a number of new candidate genes possibly involved in the interactions of wheat and *Pst*, of which 11 TDFs expressed specifically in the incompatible interaction. Resistance to stripe rust in wheat cv. Suwon11 is executed after penetration has occurred. Moreover, we also found that plant responses in compatible and incompatible interactions are qualitatively similar but quantitatively different soon after stripe rust fungus infection.

## Background

Plant disease resistance and susceptibility are governed by the combined genotypes of host and pathogen, and depend on a complex exchange of signals and responses occurring under given environmental conditions. During the long processes of host-pathogen co-evolution, plants have developed various elaborate mechanisms to ward off pathogen attack [1]. A key difference between resistant and susceptible plants is the timely recognition of the invading pathogen, and the rapid and effective activation of host defense mechanisms. The activation of defense responses in plants is initiated by host recognition of pathogen-encoded molecules called elicitors [2]. The interaction of pathogen elicitors with host receptors likely activates a signal transduction cascade that may involve protein phosphorylation, ion fluxes, reactive oxygen species (ROS), and other signaling events [3,4]. Subsequent transcriptional and/or posttranslational activation of transcription factors eventually leads to the induction of plant defense related genes [5]. In addition to eliciting primary defense responses, pathogen signals may be amplified through the generation of secondary plant signal molecules such as salicylic acid [6]. Both primary pathogen elicitors and secondary endogenous signals may trigger a diverse array of plant defense related genes, encoding glutathione S-transferases (GST), peroxidases, cell wall proteins, proteinase inhibitors, hydrolytic enzymes, pathogenesis-related (PR) proteins and phytoalexin biosynthetic enzymes [7].

At the macroscopic level, induced defense responses are frequently manifested in part as a hypersensitive response (HR), which is characterized by necrotic lesions resulting from localized host cell death at the site of infection [8]. Plant cell death occurring during the HR plays an important role in preventing the growth and spread of biotrophic pathogen into healthy tissues [9,10]. In addition to the localized HR, plants may respond to pathogen infection by activating defense responses in uninfected parts of the plant, expressing so called systemic acquired resistance (SAR) that can be long-lasting and often confers broad-based resistance to a variety of different pathogens [11,12].

Stripe rust (or yellow rust), caused by *Puccinia striiformis *Westend. f. sp. *tritici *Eriks. (*Pst*), is one of the most important diseases of wheat (*Triticum aestivum *L.) worldwide. Severe yield losses can result as a consequence of the rapid development and large-scale spread of the disease epidemic under optimal environmental conditions. Furthermore, the ability of *Pst *to form new races that can attack previously resistant cultivars, along with the capacity of fungal spores to travel long distances, can make control of stripe rust difficult. Over the last few years, epidemiological [13,14], genetic [15-18], histological [19] and molecular [20-23] studies on the disease and pathogen have been reported. The wheat-*Pst *interactions have been studied at the molecular genetics and ultrastructural levels [24,25]. Due to special features of hexaploid wheat with large and complex genome and difficulties for transformation, and of *Pst *without sexual reproduction and hard to culture on media [26], the use of most genetic and molecular techniques in studying genes involved in the wheat-*Pst *interactions has been largely limited. Thus, a global gene expression approach should be useful for elucidating the molecular mechanisms of the wheat-*Pst *interactions.

Significant progresses have been made for understanding the signaling processes involved in several plant-pathogen interactions [27]. A few studies on the wheat-rust fungus interaction have been carried out using the Wheat GeneChip^® ^[21,22,28,29]. The use of the Wheat GeneChip^® ^technique is often conditioned by known gene sequences arrayed on the chip, with limited ESTs unspecific to different wheat materials. In contrast, cDNA-amplified fragment length polymorphism (cDNA-AFLP) does not require prior sequence information and is universal for any organisms or interactions, and is, therefore, a powerful tool for identifying novel genes in non-model organisms [30,31], such as wheat [32]. As described by Bachem *et al*., cDNA-AFLP is an efficient, sensitive, and reproducible technique to detect differentially expressed genes dynamically [33].

In our previous study, we identified 186 genes likely involved in a compatible interaction between wheat (cv. Suwon 11) and *Pst *(pathotype CYR31) using the cDNA-AFLP technique [34]. A parallel study for an incompatible interaction should allow us to compare genes involved in the compatible and incompatible interactions, which should provides insights to molecular mechanisms of the different interactions of the important wheat-*Pst *pathosystem. The objective of this study was to determine wheat genes that are transcriptionally regulated in response to *Pst *infection using the cDNA-AFLP technique in a whole-genome scale. The quantitative real-time polymerase chain reaction (qRT-PCR) analysis was also used to validate the expression patterns of some important genes. Here, we report a number of transcript derived fragments (TDFs) that were found to be activated or suppressed during the incompatible interaction between wheat and *Pst*. In particular, a large number of genes encoding signal molecules were identified as early pathogen responsive genes and potential defense-related genes. Genes specifically expressed during the incompatible interaction were identified through comparing the transcription profiling in the present study with that of our previous study on compatible interaction [34].

## Results

### Infection process of stripe rust fungus and HR

**Figure 1:**
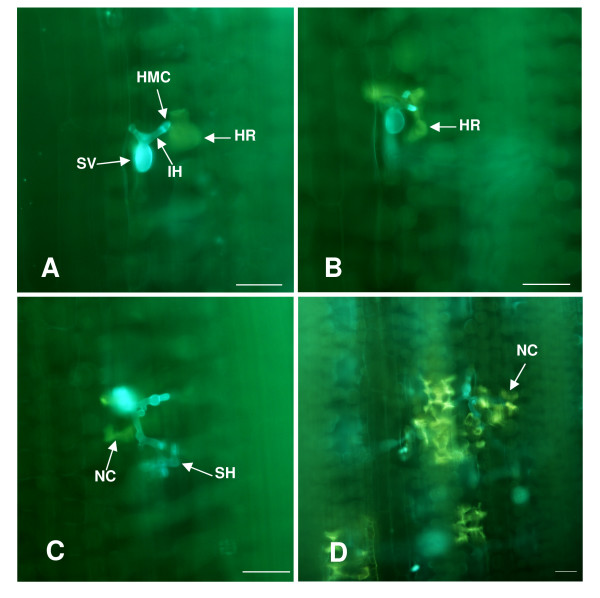
**Histology in wheat cv. 'Suwon 11' after inoculation with *P. striiformis *f. sp. *tritici *pathotype CY23 (incompatible interaction)**. (A) hypersensitive cell death (HR) was only observed in the mesophyll cells in contact with the haustorial mother cell at few infection sites, 18 hpi. (B) HR could be observed in most of infection sites, 24 hpi. (C) Second hyphae were formed and mesophll cells which around intercellular hyphae showed cell death, 72 hpi. (D) Host cell death and fungal spread inhibit at infection sites, 120 hpi. Bars = 50 μm, SV, substomatal vesicle; IH, infection hypha; HMC, haustorial mother cell; SH, secondary hyphae; NC, necrotic cell.

In the incompatible interaction, at 18 hpi when haustorial mother cells were in contact with mesophyll cells, HR was observed at the infection sites (Figure 1A). At 24 hpi, the host cells undergoing HR still looked intact (Figure 1B). With advancing incubation time, an increasing number of host cells took up HR and started to lose their original shape. Necrotic host cells could be observed at almost every infection site by 72 hpi, (Figure 1C). Up to 120 hpi, large number of host cell deceased and fungal spread were inhibited at infection sites (Figure 1D). However, in the compatible interaction, there was no indication of cell death at infection sites (data not shown). The results showed that these samples were suitable for further experiments and analyses.

### Isolation of differentially expressed genes

**Figure 2:**
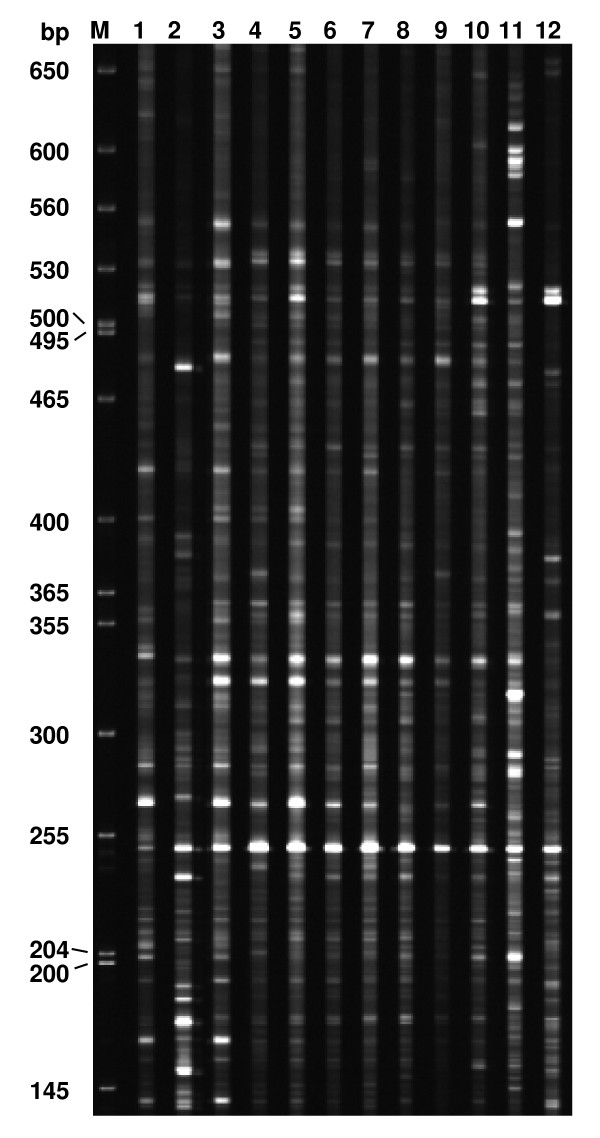
**Expression of wheat (cv. Suwon 11) genes in leaves inoculated with *Puccinia striiformis *f. sp. *tritici *(pathotype CY23) transcripts displayed by cDNA-AFLP**. An example showing selective amplification with primers MTT+TAC. Lanes 1~11 are: 6, 12, 18, 24, 36, 48, 72, 96, 120, 144 and 168 hpi, respectively; lane 12: 0 h (control plants of mock inoculation with sterile water near 0 hpi; M = molecular weight marker.

Transcript derived fragments displayed by cDNA-AFLP analysis ranged in size from 50 to 750 bp, depending on primer combinations and time points. For each of the 64 primer combinations, 55~83 bands were observed. Figure 2 showed an example of the expression patterns of the genes revealed using cDNA-AFLP with the primer pair MTT+TAC. A total of 52,992 fragments were obtained with 64 primer pairs. The cDNA-AFLP fragments were highly reproducible as the band intensities were similar from the three biological replications for each time-point. Altered expression patterns after inoculation were detected for 2,437 TDFs compared to the near 0 hpi mock-inoculation control and among the different time points, accounting for 4.6% of displayed fragments. Of the 2,437 TDFs, 1,787 were up-regulated and 650 down-regulated. A total of 300 TDFs were selected based on their intensity differences at various time points for attempted further analysis, of which 255 were recovered from gels, re-amplified, cloned and sequenced.

### Gene sequence analysis

**Figure 3:**
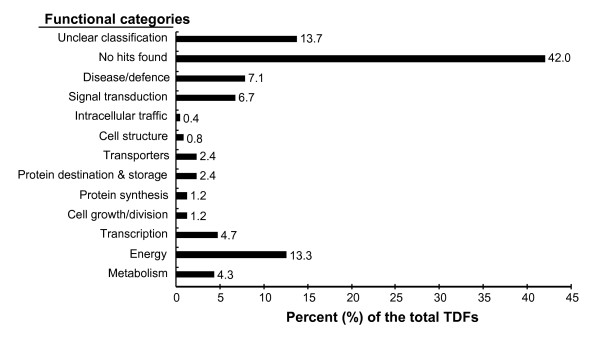
**Classification of differentially accumulated transcript derived fragments (TDFs) after inoculation with *Puccinia striiformis *f. sp. *tritici***. A total of 255 TDFs were classified based on the BLASTX homology search.

The 255 TDFs produced reliable (>100 bp) sequences. To verify the sequences for the respective bands in the cDNA-AFLP analysis, at least three clones were sequence for each re-amplified TDF and they produced an identical sequence. The sequence of each TDF was identified by similarity search using the BLASTX program against the GenBank non-redundant public sequence database. The TDF sequences were classified into functional categories based on their homology to known proteins according to Bevan's method [35]. No function could be assigned to 142 (55.7%) of the TDFs as they showed no or low sequence similarities in the database search. A group of 45 sequences (17.6%) were identified to be involved in metabolism and photosynthesis. Eighteen (7.1%) sequences shared high similarities to genes with functions in disease defense, and 17 (6.7%) sequences were found to be involved in signal transduction. The remaining 33 (12.9%) sequences were classified into groups of genes involved in transcription/transport process, protein metabolism and cell structure (Figure 3). The number of TDFs that were up- or down-regulated in each function category is summarized in Table 1. All of the 255 unigenes were submitted to the NCBI database with accession numbers assigned (see Additional File 1).

**Table 1:** The numbers of transcript derived fragments (TDFs) in some of the biological function categories showing up- or down- regulated expression pattern in wheat (cv. Suwon 11) leaves inoculated with *Puccinia striiformis *f. sp. *tritici *(pathotype CYR23)

			Expression pattern (% in category)	
			
Function group	Number	Percentage (%)	Up (%)	Down (%)
1. Sequenced	255	10.5	187 (73.3)	68 (26.7)
Metabolism	11	4.3	8 (72.7)	3 (27.3)
Energy	34	13.3	26 (76.5)	8 (23.5)
Cell growth/division	3	1.2	2 (66.7)	1 (33.3)
Transcription	12	4.7	11 (91.7)	1 (8.3)
Protein synthesis	3	1.2	3	0
Protein destination and storage	6	2.4	3 (50.0)	3 (50.0)
Transporters	6	2.4	5 (83.3)	1 (16.7)
Intracellular traffic	1	0.4	1	0
Cell structure	2	0.8	2	0
Signal transduction	17	6.7	14 (82.4)	3 (17.6)
Disease/defense	18	7.1	15 (83.3)	3 (16.7)
Unclassified	107	42.0	72 (67.3)	35 (32.7)
Unclear classification	35	13.7	25 (71.4)	10 (28.6)
2. Unsequenced	2,182	89.5	1,600 (73.3)	582 (26.7)

Total	2,437	100.0	1,787 (73.3)	650 (26.7)

The BLASTN searching of the *P. graminis *f. sp. *tritici *genome database for the 255 TDFs indicated that 19 (7.4%) of the sequenced TDFs were likely from *Pst *and 129 (50.6%) were likely from wheat, and remaining 107 (42%) were unclear about their origin because they had no hit. Of the 19 TDFs, putatively encoding ATP synthase, glycine dehydrogenase, fructose-1,6-bisphosphatase, ATP-dependent RNA helicase, T-complex protein, enolase and conserved hypothetical proteins, 11 had significant homologies (e < 1 × 10-20) to *P. graminis *f. sp. *tritici *genes. However, all of the 19 TDFs were finally determined to be from wheat through PCR amplification of the genomic DNA of the Suwon 11 wheat and CYR23 *Pst *pathotype, as 17 fragments were amplified only from the wheat, and 2 from both wheat and stripe rust pathogen but the wheat sequences were identical to those of the TDFs.

The comparative analysis between the incompatible and compatible interactions by the TBLASTN searching showed that 161 of the 255 TDFs identified in the incompatible interaction were also induced in the compatible interaction [34], indicating that the 161 TDFs are involved in the basal defense. The remaining 94 TDFs were considered to be expressed more specifically in the incompatible interaction (see Additional File 1), 43 of 94 TDFs were further analyzed by qRT-PCR.

### Validation of expression patterns by qRT- PCR analysis

**Figure 4:**
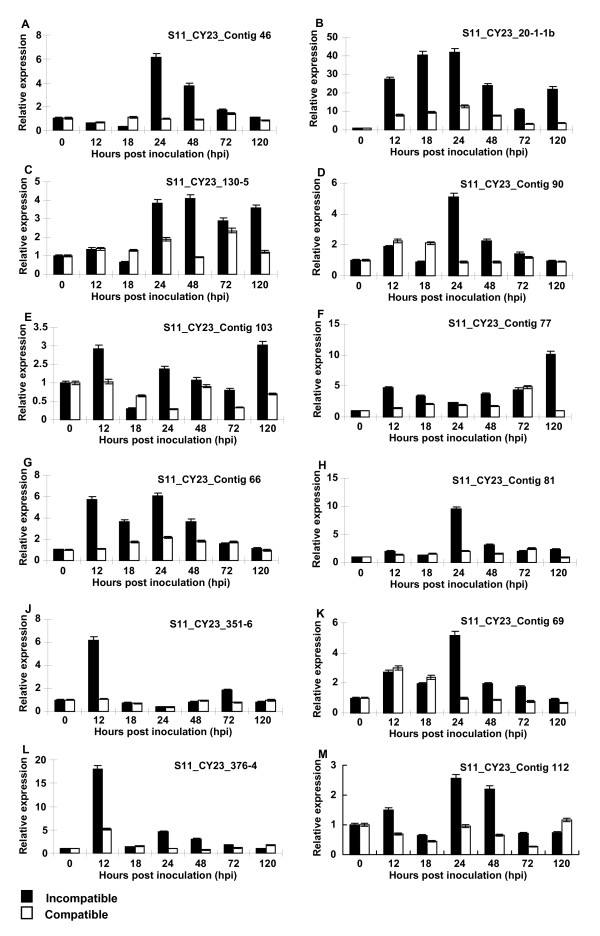
**Quantitative real-time PCR (qRT-PCR) analyses of 12 selected genes**. Leaf tissues were sampled for both inoculated and mock-inoculated plants at 12, 18, 24, 48, 72 and 120 hpi, as well as mock-inoculated near 0 hpi. Three independent biological replications were performed. Relative gene quantification was calculated by comparative ΔΔCT method. All data were normalized to the 18S rRNA expression level. The mean expression value was calculated for every transcript derived fragment (TDF) with three replications.

**Figure 5:**
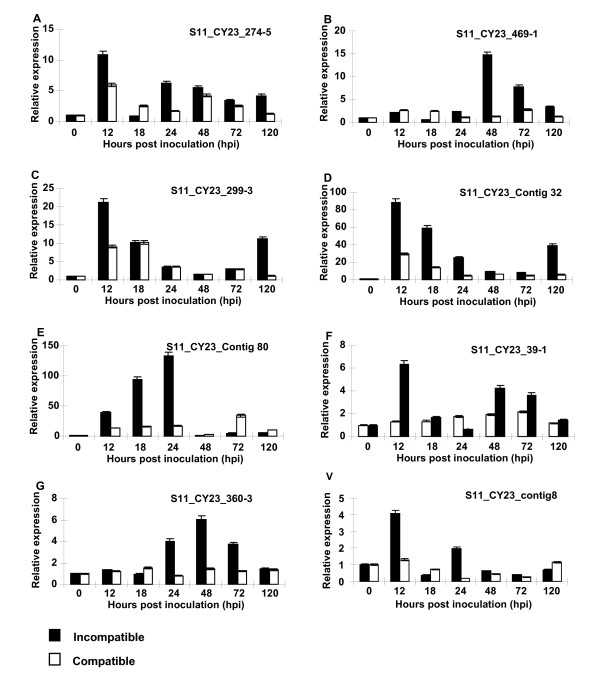
**Quantitative real-time PCR (qRT-PCR) analyses of 8 selected genes**. Leaf tissues were sampled for both inoculated and mock-inoculated plants at 12, 18, 24, 48, 72 and 120 hpi, as well as mock-inoculated near 0 hpi. Three independent biological replications were performed. Relative gene quantification was calculated by comparative ΔΔCT method. All data were normalized to the 18S rRNA expression level. The mean expression value was calculated for every transcript derived fragment (TDF) with three replications.

To validate the results of the cDNA-AFLP and comparative analyses, twenty TDFs were studied for verifying the expression patterns identified in the cDNA-AFLP study using 7 time-points (0, 12, 18, 24, 48, 72 and 120 hpi) (Figure 4, Figure 5), forty three TDFs were analyzed mainly for comparing their expressions in the incompatible and compatible interactions, and therefore, only 4 time-points (0, 12, 24 and 48 hpi) were chosen based on their major differences in the cDNA-AFLP study (Table 2). The results showed that 27 of 43 TDFs were induced in both incompatible and compatible interactions. Although in similar expression patterns, the 27 genes expressed earlier and at higher levels in the incompatible interaction than in the compatible interaction, of which 8 TDFs had expression levels great than 10 folds of the mock inoculated controls (Table 2, Figure 4, Figure 5). The transcriptional products of 5 TDFs were decreased slightly in both interactions, compared with those in the controls (Table 2). The remaining 11 TDFS were up-regulated in the incompatible interaction, but did not have significant changes or were down-regulated in the compatible interaction (Table 2).

**Table 2:** Transcript derived fragments (TDFs), which were specifically expressed in incompatible interaction by BLASTN analyzing, commonly or differentially expressed in the incompatible and compatible interactions between wheat cultivar Suwon 11 and *Puccinia striiformis *f. sp. *tritici *pathotypes CYR23 and CYR31, respectively.

	Accession	12 hpi		24 hpi		48 hpi	
		
TDF	**No**.	***I***	***C***	***I***	***C***	***I***	C
**Up-regulated in both interactions**							

S11_CY23_129-1	FG618817	+	+	-	-	+	NC

S11_CY23_Contig39	FG618708	NC	NC	+	-	NC	NC

S11_CY23_42b-2	FG618898	++	+	+	+	+	-

S11_CY23_253-1	FG618863	+	+	+	NC	+	+

S11_CY23_130-5	FG618819	NC	NC	+	+	+	NC

S11_CY23_Contig66	FG618735	++	NC	++	+	+	NC

S11_CY23_274-5*	FG618919	+++	++	++	NC	+	+

S11_CY23_299-3*	FG618916	+++	++	+	+	NC	NC

S11_CY23_90-6	FG618918	+	+	+	NC	NC	NC

S11_CY23_Contig32*	FG618701	+++	++	++	NC	+	NC

S11_CY23_Contig69	FG618738	+	+	++	NC	+	NC

S11_CY23_39-1*	FG618892	++	NC	+	NC	+	+

S11_CY23_181-4	FG618829	+	NC	+	+	NC	NC

S11_CY23_Contig7	FG618676	+	NC	-	-	NC	NC

S11_CY23_Contig58	FG618727	+	+	NC	NC	+	-

S11_CY23_Contig93	FG618762	+	NC	-	NC	-	-

S11_CY23_Contig98	FG618767	+	+	+	NC	+	NC

S11_CY23_Contig130	FG618798	+	+	+	NC	+	+

S11_CY23_349-2	FG618877	+	NC	+	+	+	NC

S11_CY23_Contig134	FG618802	-	-	+	+	NC	NC

S11_CY23_209-5	FG618844	+	+	+	NC	NC	NC

S11_CY23_257-1-3*	FG618866	+	+	+++	++	+	NC

S11_CY23_257-3	FG618869	++	+	NC	+	+	NC

S11_CY23_397p-5	FG618896	NC	NC	NC	NC	+	+

S11_CY23_376-4*	FG618889	+++	++	++	NC	+	NC

S11_CY23_257-1-5*	FG618868	+++	+	NC	NC	+	NC

S11_CY23_360-3*	FG618886	NC	NC	+	NC	++	+

**Down-regulated in both interactions**							

S11_CY23_Contig4	FG618673	NC	NC	-	-	-	NC

S11_CY23_236-3	FG618859	-	-	NC	-	NC	NC

S11_CY23_231-1	FG618855	-	-	NC	-	NC	NC

S11_CY23_Contig5	FG618674	-	-	NC	NC	-	-

S11_CY23_Contig126	FG618794	-	-	NC	NC	NC	NC

**Up in incompatible, but did not change in compatible**							

S11_CY23_193-4	FG618834	+	NC	+++	NC	+	NC

S11_CY23_128-2	FG618814	-	NC	+	NC	+	NC

S11_CY23_351-6	FG618917	++	NC	NC	NC	NC	NC

S11_CY23_469-1	FG618920	+	NC	+	NC	+++	NC

S11_CY23_397p-3	FG618895	+	NC	NC	NC	NC	NC

S11_CY23_Contig99	FG618768	NC	NC	NC	NC	+	NC

S11_CY23_148-5	FG618824	NC	NC	+	NC	NC	NC

S11_CY23_181-5	FG618830	+	NC	+	NC	NC	NC

**Up in incompatible and down in compatible**							

S11_CY23_Contig73	FG618742	NC	NC	+	-	NC	-

S11_CY23_Contig105	FG618774	NC	-	+	-	+	NC

S11_CY23_12-1-3	FG618811	+	--	+	--	NC	-


* Strongly induced in incompatible interaction

NC No changes in expression profiling

+ Transcripts >2 folds

++ Transcripts > 2 folds and < 5 folds

+++ Transcripts >10 folds

- Transcripts were repressed

The 20 genes selected to verify the cDNA-AFLP results included 8 genes (S11_CY23_contig46, S11_CY23_contig8, S11_CY23_contig69, S11_CY23_contig112, S11_CY23_contig103, S11_CY23_contig32, S11_CY23_contig80 and S11_CY23_360-3) putatively involved in disease defense, 6 genes (S11_CY23_contig66, S11_CY23_351-6, S11_CY23_contig90, S11_CY23_274-5, S11_CY23_299-3, and S11_CY23_469-1) putatively involved in signal transduction, 2 gene (S11_CY23_376-4, S11_CY23_39-1) in the "no-hit" group, and 4 genes (S11_CY23_20-1-1b, S11_CY23_130-5, S11_CY23_contig77, and S11_CY23_contig81) in other categories. All of the genes were up-regulated and their transcripts increased as early as 12 hpi, except for TDFs S11_CY23_contig46 and S11_CY23_130-5, whose expression did not increased until 24 hpi and for S11_CY23_469-1 until 48 hpi. The accumulation of transcripts of eight genes (S11_CY23_contig8, S11_CY23_39-1, S11_CY23_376-4, S11_CY23_contig103, S11_CY23_contig32, S11_CY23_351-6, S11_CY23_274-5, and S11_CY23_ 299-3) peaked at 12 hpi with *Pst*, and the others peaked at 24 hpi, except that the maximum induction of S11_CY23_469-1 and S11_CY23_130-5 transcripts occurred at 48 hpi and then steadily decreased to the original levels. TDF S11_CY23_contig77 was activated as early as 12 hpi, and followed by a slight decrease, this gene reached their maximum accumulation of transcripts at 120 hpi. For all of the 20 genes, the expression patterns of the qRT-PCR were similar to those observed in the cDNA-AFLP tests. The results showed that the cDNA-AFLP technique was more efficient in identified expressed genes and also indicated that all of the studied genes were induced by the *Pst *infection

## Discussion

Transcriptomics is a powerful approach for the global analysis of plant-pathogen interactions. Using the cDNA-AFLP technique, we observed widespread modulation of transcriptional activity, with 4.6% of all transcripts showing some form of differential expression. The gene expression patterns revealed by the cDNA-AFLP and qRT-PCR analyses were largely consistent with the physiological and biochemistry changes corresponding to the *Pst *infection events in the wheat leaf tissue.

About 73% (187) of the 255 differentially expressed genes in wheat were up-regulated during the infection process. Most of these genes peaked at 12~24 hpi, possibly reflecting the exploitation of cellular resources and/or the activation of defense responses [36,31]. The up-regulated genes were similar to that of a recent study reported by Coram *et al*. [22]. They reported that 64 genes specifically involved in the incompatible reaction between the *Yr5 *single gene line with a US Pst pathotype, PST-78, and these genes were up-regulated and peaked at 12-24 hpi. In these study, we identified 94 genes preferably induced in the incompatible interaction, also around 12-24 hpi. The most of the genes identified in both studies were characterized in the same functional categories. In contrast, Coram *et al*. identified only one gene down-regulated in the incompatible interaction [22]. Comparatively, 68 TDFs of 255 differentially expressed genes showed down-regulation in the present study. Such difference might be due to the fact that the probes of Affymetrix GeneChip were designed based on known wheat genes. Meantime, the transcriptional profiling obtained by cDNA-AFLP technique largely covered overall wheat transcriptome. Moreover, different genotypes of wheat or *Pst *pathotype, as well as different temperatures used in the tests, might also contribute to the difference. Given that genetic manipulation for *Pst *is unavailable, along with unstable wheat transformation system, the putative functions of a large number of genes identified in this study have only been predicted by bioinformatical approaches combined with altered expression patterns. Of the 255 sequenced TDFs, 113 had relatively clear functions in various categories when searching the non-redundant protein database. Thus, these genes can be valuable resources for understanding molecular changes in the incompatible interaction.

A fascinating discovery in this study is the quenching of divergent expression of *Pst*-regulated genes in both incompatible and compatible interactions in the middle stages of *Pst *infection. Similar to the results of our previous study [34], the expression of nearly all wheat genes that were differentially regulated at the early time frame returned to the levels of the mock-inoculted plants by 48 hpi. The low level of expression remained up to 120 hpi. The lack of differential gene expression at the period from 72 to 120 hpi could be because *Pst *might initiatively inhibit the early host responses in both incompatible and compatible interactions. Haustorium-forming fungi and oomycetes secrete many proteins from the haustoria into the extrahaustorial matrix during the parasitic stage of host infection, subsequently, a subset of proteins are further transported into the host cell [37]. Presumably, to establish infection, these proteins enable the pathogens to obtain nutrients, or to evade or manipulate host defenses [37,38]. It is thought that the oomycete *Phytophthora *also forms haustoria and secretes molecular signals that functions in the plant apoplast and cytoplasm to reprogram molecular host defenses [39]. The response of resistant plants at 48-72 hpi suggests that an avirulence gene is recognized by a resistance gene prior to this time, which appears to lead to a depression of host defenses. Our results may reflect that the resistance gene can be recognized by avirulence gene prior to 48 hpi in the interaction of wheat-*Pst*. Similar to our results, a study of the soybean response to Asian soybean rust (ASR) controlled by the *Rpp2 *gene also found many ASR-regulated genes responded early during the infection (6 to 36 hpi), followed by a period (24 to 72 hpi) in which expression levels returned to the mock levels and a new differentiation in gene expression during late infection (72 to 168 hpi) [40].

The development of *Pst *on resistant and susceptible wheat cv. Suwon 11 was found to be similar in urediniospore germination, appressorium formation, and penetration (foundation of substomatal vesicle, infection hypha, haustorial mother cell, and haustorium initial). However, after penetration (24 hpi), distinct differences in fungal spread between the compatible and incompatible interaction could be observed. In the compatible interaction, hyphae of CYR31 rapidly colonized host tissues intercellularly and numerous haustoria in the adjacent host cells were formed. In the incompatible interaction, the host cells showed hypersensitive cell death and the density of the intercellular hyphae and the number of haustoria were greatly reduced compared to the compatible interaction [24]. These results suggested that resistance to stripe rust in wheat cv. Suwon11 is executed after penetration has occurred. Yet, for host response to *Pst*, H_2_O_2 _accumulation was detected in host guard cells as early as 6-8 hpi [19], therefore, the perception of the *Pst *fungus by wheat and the ability of the pathogen to avoid or overcome the host's defense imply a complex, dynamic network of communication, a series of signal events should be operated before the resistance gene is expressed. Relative specifically expression of the 94 genes in the incompatible interaction and their diverse putative functions in various metabolisms support the hypothesis. However, how and when the signal is perceived by the host and transduced is still poorly understood. In this study, we focused on genes that accumulated preferentially in the incompatible reaction before 48 hpi. Dissection of these genes and their involved biochemical pathways in the future studies might provide answers to the questions.

Because the regulation of gene expression is a dynamic process, the expression profiling was presented over a time course by cDNA-AFLP, which allowed us to study the dynamic behavior of gene expression and characterize their changes over time. The induction and signal transduction of defense responses specific to the interaction require up- or down- regulation of many genes. We were primarily interested in genes whose expression might be used to distinguish incompatible from compatible interactions in wheat. A different analysis was conducted to achieve the goal by comparing gene expressions in Suwon 11 challenged with CYR23 (avirulent) or CYR31 (virulent). The comparison analysis of 255 TDFs in the incompatible interaction with those in the compatible interaction as previously described [34] showed that of the 255 transcripts induced during the incompatible interaction, 161 TDFs (63%) were also induced during the compatible interaction, and thus were classified as basal defense-related. 94 TDFs were expressed preferably in the incompatible interaction. The large proportion of TDFs were shared in both interactions, these results were similar to the reports of Coram *et al*. [22] with the same pathosystem. Coram *et al*. [22] reported 51 genes commonly induced in both incompatible and compatible interactions between wheat and *Pst*. Tao *et al*. [41] also found that plant responses in compatible and incompatible interactions are qualitatively similar but quantitatively different soon after infection. Another study of the barley response to powdery mildew controlled by the *Mla6*, *Mla13 *and *Mla1 *single resistance genes also provided evidence for a shared response between compatible and incompatible interactions up to the point of pathogen penetration [42].

Of our special interest is that 11 of 94 TDFs were up-regulated in the incompatible interaction, but did not change or were repressed in compatible interaction through the qRT-PCR validation. Of the 11 TDFs, 6 (S11_CY23_193-4, S11_CY23_128-2, S11_CY23_181-5, S11_CY23_Contig73, S11_CY23_Contig105 and S11_CY23_12-1-3) have unknown functions and 5 (S11_CY23_397p-3, S11_CY23_469-1, S11_CY23_351-6, S11_CY23_Contig99 and S11_CY23_148-5) encode Leucine Rich Repeat family protein, CBL-interacting protein kinase, Serine/threonine Kinase, ethylene-responsive RNA helicase and protein phosphatase type 2C, respectively. Protein phosphatase type 2C is a negative regulator of ABA responses. Gosti *et al*. [43] reported that suppressor mutants were more sensitive to applied ABA than the wild type and displayed increases in seed dormancy, whole-plant drought tolerance, and drought rhizogenesis intensity. However, ABA is required for plant defense. Adie *et al*. [44] measured ABA hormone levels in wild-type and JA/ET/SA/ABA-related mutants after *Pythium irregulare *infection to determine whether ABA is required for overall plant resistance. They found that ABA mutants showed an increased susceptibility to *P. irregulare *compared with the wild-type background, indicating that ABA is a positive signal involved in the activation of effective defenses against this pathogen. However, several reports showed that ABA increases susceptibility by counteracting SA-dependent defenses, and ABA-dependent priming of callose biosynthesis promotes enhanced resistance to some pathogens [45]. These results supported that ABA should have a negative effect on resistance. Our results also indicated that ABA should be expected to play a negative role in response to *Pst*. DEAD-box RNA helicases had been reported to play an important role during development and stress responses in various organisms [46-48]. Rice OsBIRH1 encoding DEAD box RNA helicase was shown to function in defense responses against pathogen and oxidative stresses [46]. STRS1 (Stress response suppressor 1) and STRS2 encoding DEAD box RNA helicases were shown to function as negative regulators of ABA-dependent and ABA-independent signaling networks [48]. Zegzouti [49] isolated an Ethylene-responsive 68 (ER68, corresponding to *Arabidopsis thaliana *RNA helicase 20), their results indicated the potential for ER68 RNA helicase activity to be involved with ethylene-regulated gene expression at either the transcriptional or post-transcriptional level. Similar to their results, S11_CY23_contig99 encoding ethylene-responsive RNA helicase, was only induced in incompatible interaction, which should attribute to resistance to *Pst*, however, the precise role played by S11_CY23_contig99 still needs to be further elucidated.

Caffeoyl-CoA O-methyltransferases (CCoAOMTs) is an important enzyme that participates in lignin biosynthesis especially in the formation of cell wall ferulic esters of plants. CCoAOMT was proposed to play a pivotal role in cell wall reinforcement during the induced disease resistance response. Lignin is often deposited at the sites of wounding or pathogen invasion, which may provide a physical barrier for protection of adjacent tissues from further damage. In the previous study [24], immunogold localization of lignin revealed a markedly higher labeling density in host cell walls of the infected wheat leaves of the resistant cultivar than in cell walls of the infected wheat leaves of the susceptible cultivar. In this study, S11_CY23_360-3 encoding CCoAOMT, induced at 24 hpi, and peaked at 48 hpi in the incompatible interaction, the transcripts accumulations occurred after the resistance gene was triggered, which indicated that lignification appears to be also an active resistance mechanism in the wheat-*Pst *panthosystem.

Suwon 11 showed HR to CY23 infection. Plant cells involved in the HR generate an oxidative burst by producing reactive oxygen species (ROS), superoxide anions, hydrogen peroxide, hydroxyl radicals, and nitrous oxide [50]. Peroxidases were thought to play an important role in ROS production [51,52]. In the present study, two genes (S11_CY23_contig32 and S11_CY23_ contig46) were predicted to encode peroxidase and peroxisomal membrane protein, respectively. Their transcripts peaked as early as 12 and 24 hpi, respectively. Our gene expression data were coincident with the previous report of a rapid increase of O_2_^-- ^and H_2_O_2 _at infection sites and a strong accumulation of H_2_O_2 _in mesophyll cells from 12~24 hpi using histochemical staining in the Suwon 11 leaves inoculated by CYR23 [19]. In contrast, O_2_^- ^and H_2_O_2 _could not be detected in most of the infection sites in the compatible interaction between Suwon 11 and CYR31. In the present study, we found that the expression of these two genes were much less in the compatible interaction than in the incompatible interaction.

Several studies have provided evidence supporting that the PR-5 protein plays an active role in resistance mechanisms in cereals [53-56]. Transcripts of four PR protein genes were analyzed during *Fusarium graminearum *infection, with *PR-5 *transcript accumulated as early as 6 to 12 hpi and peaked at 48 hpi [56]. We also found S11_CY23_112 homologous to a wheat PR-5-like protein gene. S11_CY23_112 transcript accumulated strongly at 24-48 hpi. A stronger induction of this gene could be observed in the incompatible interaction than the compatible interaction, suggesting a general role of this protein in wheat resistance to stripe rust. Similarly, gene S11_CY23_274-5 from wheat was deduced to encode a receptor related to antifungal PR proteins. The predicted protein contained an extracellular domain related to the PR 5 protein, a central transmembrane spanning domain, and an intracellular protein serine/threonine kinase. Wang *et al*. isolated a *PR5K *gene from *Arabidopsis thaliana *and found that *PR5K *transcript accumulated at low levels in all tissues examined [57]. They suggested a possible interaction of PR5K with common or related microbial targets. Nevertheless, the interrelation of PR5K and PR5 protein during the interaction between wheat and *Pst *need further studies.

Protein kinase is known to play a central role in signaling during pathogen recognition and the subsequent activation of plant defense mechanisms [58]. We identified six TDFs encoding different protein kinases. S11_CY23_contig90 was highly homologous to the Arabidopsis *MKP1 *gene, which was showed to be induced at the transcriptional level during the interaction of wheat-*Pst*. The Arabidopsis genome contains 20 genes encoding mitogen-activated protein kinases (MAPKs), which interact with MKP1 [59]. Using expression profiling, a specific group of genes that probably represent targets of MKP1 regulation was also identified [59]. Surprisingly, the identity of these genes and interacting MAPKs suggested involvement of MKP1 in salt stress responses. Indeed, *mkp1 *plants have increased resistance to salinity [60]. Accordingly, the gene S11_CY23_contig90 may play a role in the integration and fine-tuning of plant responses to stripe rust pathogen challenging.

This study uncovered a number of new candidate genes possibly involved in the interactions of wheat and *Pst*. More than 42% of the sequenced TDFs had no homologous sequences in the EST databases. De Torres *et al*. [61] reported that the plant response to pathogen challenge evoked a large number of transcriptomic components not yet present in EST libraries. Over 60% of the differentially regulated transcripts in their cDNA-AFLP were absent from standard 8,200 feature Affymetrix Gene Chips [61]. Therefore, cDNA-AFLP analysis is a suitable tool for discovering new potential genes that are differentially expressed during the wheat-*Pst *interactions. Because most of the molecular mechanisms involved in the pathosystem interactions are yet to be determined, the large number of TDFs identified in this study will serve as candidates for further studies to determine their functions and dissect the molecular networks involved in the plant-pathogen interactions.

## Conclusion

In this study, we have obtained a broad overview of the behaviour of the wheat transcriptomes to the stripe rust fungus and this has provided many interesting clues to the interaction of the wheat and *Pst*. We have also seen the differences between the incompatible (resistant) and compatible (susceptible) interaction. With regard to many genes, we observed patterns of transcript accumulation that reflect those observed by other groups in their studies. However, as a consequence of this study, we have also made observations that give additional insight in the complexities of the interactions that occur in both interactions, and uncovered a number of new candidate genes possibly involved in the interactions of wheat and *Pst*. Especially, 11 TDFs expressed specifically in the incompatible interaction were observed, and these genes should play important roles in the interaction of wheat and *Pst*. However, how and when they function in the infection process, need to be further studied.

## Methods

### Plant materials and inoculation

Wheat genotype Suwon 11 and *Pst *pathotype CYR23 were used for the cDNA-AFLP analysis of an incompatible interaction. For the qRT-PCR analysis of candidate genes, pathotype CYR31 was used with Suwon 11 to form a compatible interaction as in the previous study [34]. Suwon 11 contains *YrSu *that provides seedling resistance against stripe rust, [62,63] showing a typical HR when being challenged by CYR23. Plants were grown, inoculated and maintained following the procedures and conditions described by Kang & Li [64]. Control plants corresponding to each time point were brushed with sterile water, referred to as mock inoculation. Samples of the mock inoculated leaves taken just after water-inoculation (near 0 hpi) were treated as the initial control and those from mock-inoculated leaves at each time point served as the control for that time point. Leaf tissues were harvested at 6, 12, 18, 24, 36, 48, 72, 96, 120, 144 and 168 h post-inoculation (hpi) and quickly frozen in liquid nitrogen and stored at -80°C prior to total RNA extraction. The time points were selected based on the microscopic study of the incompatible interaction between Suwon 11 and pathotype CYR23 or CYR31 [19]. Plants were rated for symptom development 15 days after inoculation. Three biological replications were performed independently for each time point.

### Histological observation of stripe rust fungus

To ensure the suitability of leaf samples for incompatible and compatible interactions, detection of stripe rust fungus infection process in different interactions was carried out using the Calcofluor White (Sigma Co., USA.) staining method as described by Kang *et al*. [65].

For microscopy, staining hyphae and autofluorescence of attacked mesophyll cells was observed using a fluorescence microscopy (excitation filter 485 nm, dichromic mirror 510 nm, barrier filter 520 nm). All microscopical examinations were done with an Olympus BX-51 microscope (Olympus Corporation, Japan). At least 50 penetration sites on each of four leaf specimens per treatment were scored.

### cDNA-AFLP analysis and TDFs isolation

Total RNA was isolated from about 200 mg of the frozen wheat leaves using the Trizol™reagent (Invitrogen, Carlsbad, CA, USA) according to the manufacture's protocol. Twenty micrograms of total RNA was used initially for the first strand synthesis, followed by the second strand synthesis using the SMART™ PCR cDNA Synthesis Kit (Clontech, Mountain View, CA, USA) following the manufacturer's instruction. About 100 ng of double-stranded cDNA was subjected to standard AFLP template production. cDNA-AFLP analysis was performed with 64 primer combinations (see Additional File 2) using the IRDye^® ^Fluorescent 800 AFLP expression analysis kit (LI-COR) and TDFs were isolated with protocols as previously described [34].

### Sequence analysis

In order to efficiently analyze the large-scale EST data, a local stand-alone EST analysis platform was set up with the Linux operation system described by Wang *et al*. [34]. In addition, the wheat stem rust pathogen (*P. graminis *f. sp. *tritici*) whole genome database http://www.broad.mit.edu/annotation/genome/puccinia_graminis was used for the BLASTN searching to determine possible *Pst *genes.

In order to compare TDFs between the incompatible and compatible reactions, all 186 uniseqs from the previous compatible interaction [34] were formatted to form one local compatible database using the command of "formatdb" in the BLAST system. Then we did the TBLASTN seaching against local compatible database for all 255 uniseqs identified in the present study from the incompatible interaction as query sequences using an E-value of 1e-20 as the high stringent cut-off point.

### Origin confirmation for genes homologous to the stem rust pathogen

Genomic DNA was extracted from the urediniospores of CYR23 and leaves of Suwon 11 according to the protocol described by Wang *et al*. [66]. Specific primers for possible *Pst *genes were designed using the software of Primer Premier 5.0. Standard PCR amplification was performed separately with genomic DNA from the urediniospores and wheat leaves as templates. The PCR products were run on an agarose gel along with a molecular size marker (DL2000, TaKaRa Biotechnology Co., Ltd), followed by sequencing analyses of all amplified bands.

### qRT-PCR and data analyses

Leaf tissues challenged by CYR23 or CYR31 were sampled at 12, 18, 24, 48, 72 and 120 hpi, as well as samplings of the control plants mock-inoculated with sterile water at each of the corresponding time points, and near 0 hpi for the mock-inoculated plants. Three independent biological replications were performed for both inoculated and control plants. Primer design, reverse transcription, and qRT-PCR reaction were conducted as described in our previous study [34]. To standardize the data, the amount of target gene transcript was normalized over the constitutive abundance of wheat 18S rRNA (GenBank accession no. AY049040). All reactions were performed in triplicate, including three non-template as the negative controls. Quantification of gene expression was performed using a 7500 Real-Time PCR System (Applied Biosystems, Forst City, CA). Dissociation curves were generated for each reaction to ensure specific amplification. Threshold values (CT) generated from the ABI PRISM 7500 Software Tool (Applied Biosystems, Foster City, CA, USA) were employed to quantify relative gene expression using the comparative 2^-ΔΔCT ^method [67].

## Authors' contributions

XJW: designed experiments, analyzed data and wrote manuscript. WL: conducted qRT-PCR and collected and analyzed data. CLT: analyzed data and prepared manuscript. YLD: provided assistance in various experiments. JBM: conducted bioinformatical analysis. XLH and GRW: prepared samples and collected data. LLH: coordinated the experiments and data analyses. XMC: provided advices for experiments and revised manuscript. ZSK: conceived the project, designed the experiments and wrote manuscript. All authors read and approved the final manuscript.

## Supplementary Material

Additional file 1 **Two hundred fifty-five sequenced transcript derived fragments (TDFs) from *Puccinia striiformis *f. sp. *tritici *(pathotype CY23) infected wheat (cv. Suwon 11) leaves with altered expression patterns detected in the incompatible interaction using cDNA-AFLP and their closest matches in the GenBank database**. containing Function group and TDF, Accession No., Size, Closest to database match, E-value, Expression and Primer pair. The number of TDFs in each category and the total number of TDFs are indicated in the parentheses and separated by "/".The TDFs marked with "*" were only found in the incompatible interaction when compared with the TDFs of the compatible interaction of the same wheat genotype, Suwon 11, with *Pst *pathotype CYR31 (See Reference No. [34]) using the BLASTN analysis; and those without "*" were common in the incompatible and compatible interactions. Click here for file

Additional file 2 **Selective primers used in cDNA-AFLP**. displaying primers used for cDNA-AFLP. Click here for file
