# Supplementary material for: Differential gene expression in incompatible interaction between wheat and stripe rust fungus revealed by cDNA-AFLP and comparison to compatible interaction
Source: BMC Plant Biol. 2010 Jan 12;10:9. doi: 10.1186/1471-2229-10-9 (PMC2817678; doi:10.1186/1471-2229-10-9)
Supplement: Additional file 1 — Two hundred fifty-five sequenced transcript derived fragments (TDFs) from Puccinia striiformis f. sp. tritici (pathotype CY23) infected wheat (cv. Suwon 11) leaves with altered expression patterns detected in the incompatible interaction using cDNA-AFLP and their closest matches in the GenBank database. containing Function group and TDF, Accession No., Size, Closest to database match, E-value, Expression and Primer pair. The number of TDFs in each category and the total number of TDFs are indicated in the parentheses and separated by "/".The TDFs marked with "*" were only found in the incompatible interaction when compared with the TDFs of the compatible interaction of the same wheat genotype, Suwon 11, with Pst pathotype CYR31 (See Reference No. [34]) using the BLASTN analysis; and those without "*" were common in the incompatible and compatible interactions. [file 1471-2229-10-9-S1.DOC]

| **Additional File 1.** Two hundred fifty-five sequenced transcript derived fragments (TDFs) from *Puccinia striiformis* f. sp. *tritici* (pathotype CY23) infected wheat (cv. Suwon 11) leaves with altered expression patterns detected in the incompatible interaction using cDNA-AFLP and their closest matches in the GenBank database | | | | | | | | |
| --- | --- | --- | --- | --- | --- | --- | --- | --- |
| Function group and TDF^a^ | Accession No. | Size  (bp) | Closest to database match | | E-value | Expression | | Primer pair |
| *1. Metabolism* (11/255) | | | | | |  | | |
| S11_CY23_Contig17* | FG618686 | 368 | emb|CAJ01706.1|S-adenosyl-L-homocysteine hydrolase [*Hordeum vulgare* ] | | 2.00E-16 | Up | | MAC/TTG |
| S11_CY23_Contig18* | FG618687 | 371 | emb|CAJ01706.1|S-adenosyl-L-homocysteine hydrolase [*Hordeum vulgare*] | | 6.00E-16 | Up | | MAC/TTG |
| S11_CY23_Contig95* | FG618764 | 214 | ref|NP_916022.1| pyrophosphate- fructose- 6- phosphate phosphotransferase [*Oryza sativa*] | | 7.00E-29 | Up | | MTG/TAG |
| S11_CY23_35-3* | FG618876 | 167 | ref|XP_469653.1| glycine hydroxymethyl transferase [*Oryza sativa*] | | 5.00E-13 | Down | | MAC/TGA |
| S11_CY23_243-5 | FG618861 | 341 | gb|AAL58883.1|methylthioadenosine/S–adenosyl homocysteine nucleosidase [*Oryza sativa*] | | 4.00E-19 | Up | | MTG/TTG |
| S11_CY23_242-2* | FG618860 | 328 | gb|ABF96135.1|Peptidylprolyl isomerase PASTICCI NO1 [*Oryza sativa*] | | 5.00E-49 | Up | | MTG/TTG |
| S11_CY23_233-1-5 | FG618857 | 292 | ref|XP_464920.1| Peroxisomal fatty acid beta- oxidation multifunctional protein [*Oryza sativa*] | | 8.00E-38 | Down | | MTG/TTG |
| S11_CY23_231-1* | FG618856 | 228 | gb|AAP83928.1| Rubisco activase beta form precursor | | 8.00E-28 | Down | | MTG/TTG |
| S11_CY23_Contig19 | FG618688 | 403 | gb|AAL58883.1|methylthioadenosine/S-adenosyl homo- cysteine nucleosidase [*Oryza sativa*] | | 2.00E-19 | Up | | MAC/TTG |
| S11_CY23_Contig29 | FG618698 | 380 | gb|AAB82711.1|glycine decarboxylase P subunit [*Tritordeum sp.*] | | 1.00E-59 | Up | | MTG/TAG |
| S11_CY23_Contig68 | FG618737 | 337 | gb|AAL58883.1|methylthioadenosine/S-adenosyl homo- cysteine nucleosidase [*Oryza sativa*] | | 2.00E-19 | Up | | MTG/TTG |
| *2. Energy* (34 /255) | | | | | |  | | |
| S11_CY23_Contig35 | FG618704 | 242 | gb|AAD34735.1| photosystem Q (B) protein precursor [*Poaannua*] | | 1.00E-15 | Down | | MTC/TCA |
| S11_CY23_Contig1 | FG618670 | 378 | emb|CAA25058.1| ribulosebisphosphate carboxylase [*Triticum aestivum*] | | 3.00E-07 | Up | | MTC/TAC |
| S11_CY23_Contig11* | FG618680 | 349 | gb|AAP72270.1| ribulose-1,5-bisphosphate carboxylase activase [*Triticum aestivum*] | | 1.00E-62 | Up | | MAC/TTG |
| S11_CY23_Contig14 | FG618683 | 317 | gb|AAV33287.1|ribulose-1,5-bisphosphatecar-boxylase /oxygenase [*Brachypodiumpinnatum*] | | 1.00E-45 | Up | | MGT/TAC |
| S11_CY23_Contig16 | FG618685 | 375 | gb|AAP72270.1| ribulose-1,5-bisphosphate carboxylase activase [*Triticum aestivum*] | | 3.00E-55 | Up | | MAC/TGA |
| S11_CY23_Contig30 | FG618699 | 526 | |gb|AAU04826.1| ribulose-1,5-bisphosphate carboxylase/ oxygenase [*Stenostachyslaevis*] | | 1.00E-86 | Up | | MGT/TAC |
| S11_CY23_Contig41* | FG618710 | 260 | gb|AAV33293.1| ribulose-1,5-bisphosphatecar-boxylase/ oxygenase [*Molinia caerule*a] | | 2.00E-40 | Up | | MTC/TAC |
| S11_CY23_Contig20* | FG618689 | 326 | ref|NP_0 43032.1| ATP synthase CF1 beta chain [Zea *mays*] | | 6.00E-06 | Down | | MAC/TTG |
| S11_CY23_Contig22* | FG618691 | 195 | gb|AAN32609.1| photosystem II D2 protein [*Yucca* *glauca*] | | 4.00E-20 | Down | | MAC/TTG |
| S11_CY23_Contig67 | FG618736 | 339 | gb|AAV31343.1| chloroplast ribosomal proteinL1 [*Oryza sativa*] | | 1.00E-49 | Up | | MTG/TTG |
| S11_CY23_Contig50 | FG618719 | 392 | pir||T02955 cytochrome P450 monooxygenase [*maize*] | | 9.00E-26 | Up | | MGT/TAC |
| S11_CY23_Contig77 | FG618746 | 638 | dbj|BAB33421.1| probable cytochrome P450 monooxygenase [*maize*] | | 2.00E-28 | Up | | MTG/TTG |
| S11_CY23_Contig51* | FG618720 | 582 | gb|AAD34735.1| photosystem Q (B) protein precursor [*Poaannua*] | | 6.00E-97 | Up | | MTC/TAC |
| S11_CY23_Contig55 | FG618724 | 305 | emb|CAB46084.1| fructose-1,6-bisphosphatase [*Pisums-* *ativum*] | | 3.00E-44 | Up | | MTG/TTG |
| S11_CY23_Contig65* | FG618734 | 233 | sp|Q42695| RuBisCO large subunit-binding protein subunit *[Arabidopsis thaliana*] | | 1.00E-15 | Up | | MTG/TTG |
| S11_CY23_Contig72 | FG618741 | 368 | gb|AAG04943.1|cytochrome oxidase subunit [*Pseudomonas aeruginos*a] | | 2.00E-55 | Up | | MTG/TTG |
| S11_CY23_Contig88 | FG618757 | 219 | emb|CAA44888.1| chlorophyll a/b binding protein precursor [*Zea may*s] | | 5.00E-08 | Down | | MTG/TAG |
| S11_CY23_Contig78 | FG618747 | 162 | gb|AAM88439.1| Rieske Fe-S precursor protein[*Triticum aestivum*] | | 1.00E-08 | Up | | MGT/TAC |
| S11_CY23_Contig114 | FG618783 | 411 | ref|XP_478377.1| ATP synthase gamma chain [*Oryza* *sativa*] | | 6.00E-39 | Down | | MTG/TAG |
| S11_CY23_Contig83 | FG618752 | 273 | ref|NP_910644.1| putative vacuolar ATP synthase subunit [*Oryza sativa*] | | 2.00E-32 | Up | | MTC/TCA |
| S11_CY23_Contig106* | FG618775 | 227 | emb|CAA46804.1| H(+)-transporting ATP synthase [*Zea* *mays*] | | 7.00E-18 | Up | | MTG/TAG |
| S11_CY23_Contig110 | FG618779 | 401 | sp|P40880| Carbonic anhydrase, chloroplast precursor [*Oryza sativa*] | | 2.00E-46 | Up | | MTG/TAG |
| S11_CY23_Contig133 | FG618801 | 435 | sp|P40880|Carbonic anhydrase, chloroplast precursor [*Oryza sativa*] | | 3.00E-19 | Up | | MAC/TGA |
| S11_CY23_349-2* | FG618875 | 169 | gb|AAL59917.1| enolase [*Arabidopsis thaliana*] | | 7.00E-16 | Up | | MTG/TAG |
| S11_CY23_231-1* | FG618856 | 352 | gb|AAP83928.1| Rubisco activase beta form precursor [*Deschampsia antarctica*] | | 6.00E-54 | Down | | MTG/TTG |
| S11_CY23_199-4 | FG618836 | 348 | emb|CAA25058.1| ribulosebisphosphate carboxylase [*Triticum aestivum*] | | 7.00E-07 | Up | | MTC/TAC |
| S11_CY23_205-1-5* | FG618845 | 170 | gemb|CAD54631.1| NADP-dependant malate dehydro- genase [*Oplismenus compositus*] | | 2.00E-18 | Up | | MTC/TAC |
| S11_CY23_213-1-1 | FG618847 | 305 | emb|CAB46084.1| fructose-1,6-bisphosphatase [*Pisumsativum*] | | 3.00E-44 | Up | | MTC/TAC |
| S11_CY23_382-1 | FG618888 | 169 | emb|CAA25058.1| ribulosebisphosphate carboxylase [*Triticum aestivum*] | | 2.00E-07 | Up | | MTG/TAG |
| S11_CY23_378-2 | FG618887 | 405 | sp|P40880| Carbonic anhydrase, chloroplast precursor [*Oryza sativa*] | | 2.00E-47 | Up | | MTG/TAG |
| S11_CY23_187-1-5 | FG618832 | 221 | sp|P40880| Carbonic anhydrase, chloroplast precursor [*Oryza sativa*] | | 2.00E-22 | Up | | MTC/TAC |
| S11_CY23_357-3* | FG618882 | 221 | ref|NP_916022.1| pyrophosphate- fructose-6- phosphate1 phospho transferase [*Oryza sativa*] | | 2.00E-14 | Down | | MTG/TAG |
| S11_CY23_42b-1* | FG618894 | 214 | emb|CAA25114.1| ATPase, beta subunit [*Hordeum* *vulgare*] | | 6.00E-28 | Down | | MGT/TAC |
| S11_CY23_Contig76 | FG618745 | 416 | ref|XP_478627.1| Oxygen-evolving enhancer protein [*Oryza sativa*] | | 5.00E-22 | Up | | MTC/TAC |
| 3. *Cell growth/division* (3/255) | | | | | |  | | |
| S11_CY23_129-1* | FG618817 | 359 | dbj|BAC78562.1| thiamine biosynthetic enzyme [*Oryza* *sativa*] | | 6.00E-19 | Up | | MAC/TTG |
| S11_CY23_359-3 | FG618883 | 196 | emb|CAC59976.1| pollen signalling protein with adenylylcyclase activity [*Zea mays*] | | 5.00E-18 | Down | | MTG/TAG |
| S11_CY23_Contig39* | FG618708 | 250 | dbj|BAD36295.1| RAD23 protein [*Oryza sativa*] | | 9.00E-31 | Up | | MTC/TAC |
| *4. Transcription* (12/255) | | | | | |  | | |
| S11_CY23_Contig27 | FG618696 | 369 | gb|ABA97622.2| RNA binding protein [*Oryza sativa*] | | 2.00E-56 | Down | | MAC/TTG |
| S11_CY23_Contig107 | FG618776 | 203 | gb|AAK13589.1| rRNA intron-encoded homing endo- nuclease [*Oryza sativa*] | | 4.00E-19 | Up | | MGT/TAC |
| S11_CY23_Contig125 | FG618793 | 394 | gb|AAK13589.1| rRNA intron-encoded homing endo- nuclease[*Oryza sativa*] | | 1.00E-29 | Up | | MTC/TAC |
| S11_CY23_Contig136 | FG618804 | 426 | gb|ABA97622.2| RNA binding protein [*Oryza sativa*] | | 9.00E-63 | Up | | MGT/TAC |
| S11_CY23_44-2-1b | FG618898 | 202 | gb|AAK13589.1| rRNA intron-encoded homing endo- nuclease [*Oryza sativa*] | | 4.00E-19 | Up | | MGT/TAC |
| S11_CY23_42b-2* | FG618895 | 455 | gb|AAT83955.1| MarR-family transcriptional regulator [*Propionibacterium acnes*] | | 1.00E-66 | Up | | MGT/TAC |
| S11_CY23_253-1* | FG618863 | 561 | gb|AAM97452.1| RNA polymerase alpha subunit [*Elymusvirginicus*] | | 7.00E-97 | Up | | MTG/TTG |
| S11_CY23_360-3* | FG618886 | 173 | gb|AAT68022.1| caffeoyl-CoA O-methyl transferase [*Oryza sativa*] | | 1.00E-19 | Up | | MTG/TAG |
| S11_CY23_Contig99* | FG618768 | 242 | ref|NP_913140.1| ethylene-responsive RNA helicase [*Oryza sativa*] | | 1.00E-19 | Up | | MTG/TAG |
| S11_CY23_130-5* | FG618819 | 317 | emb|CAA90651.1| elongation factor 1-alpha [*Hordeumvulgare*] | | 1.00E-32 | Up | | MAC/TTG |
| S11_CY23_205-1-4* | FG618844 | 169 | ref|XP_479160.1| oligouridylate binding protein [*Oryza sativa*] | | 8.00E-06 | Up | | MTC/TAC |
| S11_CY23_203-2 | FG618841 | 348 | gb|AAK13589.1| rRNA intron-encoded homing endo- nuclease [*Oryza sativa*] | | 1.00E-25 | Up | | MTC/TAC |
| 5. *Protein synthesis* (3/255) | | | | | |  | | |
| S11_CY23_2-2b* | FG618837 | 287 | dbj|BAE20413.1| aspartic proteinase [*Triticum* *aestivum*] | | 3.00E-45 | Up | MGT/TAC | |
| S11_CY23_397p-5* | FG618893 | 314 | emb|CAA46810.1| cathepsin B [*Triticum* *aestivum*] | | 4.00E-52 | Up | MTG/TAG | |
| S11_CY23_257-1-4* | FG618867 | 179 | ref|XP_473432.1| OSJNBa0010H02.6 [*Oryza sativa*] | | 3.00E-17 | Up | MTG/TTG | |
| 6. *Protein destination and storage* (6/255) | | | | | |  | | |
| S11_CY23_Contig26 | FG618695 | 228 | emb|CAA63139.1| aminolevulinate dehydratase [*Hordeumvulgare*] | 2.00E-12 | | Up | MTC/TCA | |
| S11_CY23_Contig63 | FG618732 | 225 | gb|AAL77200.1| ubiquitin [*Oryza sativa*] | 7.00E-23 | | Down | MGT/TAC | |
| S11_CY23_Contig81 | FG618750 | 427 | gb|AAL77200.1| ubiquitin [*Oryza sativa*] | 2.00E-23 | | Up | MGT/TAC | |
| S11_CY23_Contig104 | FG618773 | 263 | gb|AAL25813.1| polyubiquitin [*Prunus avium*] | 2.00E-22 | | Up | MTG/TAG | |
| S11_CY23_48-3 | FG618903 | 230 | gb|AAL77200.1| ubiquitin [Oryza sativa] | 2.00E-22 | | Down | MAC/TGA | |
| S11_CY23_Contig116* | FG618785 | 313 | ref|XP_472295.1| OSJNBa0055C08.1 [*Oryza sativa*] | 3.00E-20 | | Down | MTC/TAC | |
| *7. Transporters* (6/255) | | | | | |  | | |
| S11_CY23_Contig25 | FG618694 | 233 | ref|XP_473919.1| OSJNBa0058K23.17 [*Oryza sativa*] | 4.00E-06 | | Up | MAC/TGA | |
| S11_CY23_Contig102 | FG618771 | 208 | ref|XP_473919.1| OSJNBa0058K23.17 [*Oryza sativa*] | 6.00E-06 | | Up | MTC/TAC | |
| S11_CY23_228-3 | FG618854 | 211 | ref|XP_473919.1| OSJNBa0058K23.17 [*Oryza sativa*] | 6.00E-06 | | Up | MTG/TTG | |
| S11_CY23_Contig52 | FG618721 | 167 | ref|XP_474204.1|OSJNBa0011F23.20 [*Oryza sativa*] | 4.00E-16 | | Up | MTC/TAC | |
| S11_CY23_125-1 | FG618813 | 285 | ref|XP_474204.1| OSJNBa0011F23.20 [*Oryza sativa*] | 4.00E-16 | | Up | MAC/TTG | |
| S11_CY23_55-1b | FG618909 | 195 | ref|XP_474204.1| OSJNBa0011F23.20 [*Oryza sativa*] | 1.00E-15 | | Down | MGT/TAC | |
| *8. Intracellular traffic* (1/255) | | | | | |  | | |
| S11_CY23_20-1-1b | FG618839 | 306 | ref|XP_463067.1| peroxisomal membrane protein [*Oryza sativa*] | 7.00E-37 | | Up | MGT/TAC | |
| *9. Cell structure* (2/255) | | | | | |  | | |
| S11_CY23_Contig75 | FG618744 | 405 | ref|ZP_00817021.1| flagellin type B [*Marinobactera quaeolei* ] | 3.00E-17 | | Up | MAC/TGA | |
| S11_CY23_Contig82 | FG618751 | 269 | ref|XP_475020.1| OSJNBb0093G06.1 [*Oryza sativa*] | 1.00E-29 | | Up | MTC/TCA | |
| *10. Signal transduction* (17/255) | | | | | |  | | |
| S11_CY23_145-5* | FG618822 | 245 | dbj|BAD38042.1| protein phosphatase 2C [*Oryza sativa*] | 7.00E-10 | | Up | MAC/TTG | |
| S11_CY23_148-5* | FG618824 | 239 | ref|XP_478310.1| protein phosphatase type 2C[*Oryza sativa*] | 4.00E-15 | | Up | MCA/TGT | |
| S11_CY23_Contig4* | FG618673 | 563 | gb|ABA96592.1| Protein-tyrosine phosphatase containing protein [*Oryza sativa*] | 2.00E-57 | | Down | MTC/TCA | |
| S11_CY23_Contig89 | FG618758 | 153 | ref|XP_467296.1| phosphoribulokinase precursor [*Oryza sativa*] | 5.00E-16 | | Down | MGT/TAC | |
| S11_CY23_Contig90 | FG618759 | 171 | emb|CAD42640.1| MAPKK kinase [*Hordeum vulgaresubsp*] | 3.00E-17 | | Up | MTC/TAC | |
| S11_CY23_249-2 | FG618862 | 407 | ref|XP_463768.1| tetratricopeptide repeat(TPR) –containing protein [*Oryza sativa*] | 7.00E-24 | | Up | MCT/TTC | |
| S11_CY23_236-3* | FG618859 | 327 | ref|XP_467523.1| putative WD-40 repeat protein [*Oryza sativa*] | 2.00E-41 | | Up | MGA/TCT | |
| S11_CY23_Contig66* | FG618735 | 340 | ref|XP_467523.1| putative WD-40 repeat protein [Oryza sativa] | 1.00E-51 | | Up | MTG/TTG | |
| S11_CY23_Contig121 | FG618790 | 371 | dbj|BAD37980.1| phosphatase-like [*Oryza sativa*] | 3.00E-25 | | Up | MTC/TCA | |
| S11_CY23_Contig96 | FG618765 | 223 | ref|NP_914288.1| P0458E05.17 [*Oryza sativa*] | 7.00E-21 | | Up | MTG/TAG | |
| S11_CY23_213-1 | FG618849 | 307 | gb|AAM76682.1| peroxidase [*Triticum aestivum*] | 2.00E-41 | | Up | MTC/TAC | |
| S11_CY23_8-3b | FG618914 | 648 | gb|ABF95153.1| Variant SH3 domain containing protein [*Oryza sativa*] | 1.00E-62 | | Down | MGT/TAC | |
| S11_CY23_274-5* | FG618919 | 716 | ref|XP_550059.1| receptor serine/ threonine kinase [*Oryza sativa*] | 1.00E-11 | | Up | MGA/TTC | |
| S11_CY23_469-1* | FG618920 | 621 | [sp|Q7X996.1| CBL-interacting protein kinase [Sorghum bicolor]](http://www.ncbi.nlm.nih.gov/entrez/query.fcgi?cmd=Retrieve&db=Protein&list_uids=75326963&dopt=GenPept&RID=8A8DUJW401N&log$=protalign&blast_rank=1) | 5.00E-45 | | Up | MAG/TCT | |
| S11_CY23_299-3* | FG618916 | 633 | gb|AAX19515.1| serine/threonine protein kinase [*Triticum aestivum*] | 1.00E-61 | | Up | MCT/TCT | |
| S11_CY23_351-6* | FG618917 | 524 | ref|XP_467322.1| serine/threonine- protein kinase [*Oryza sativa*] | 9.00E-17 | | Up | MAG/TGT | |
| S11_CY23_90-6* | FG618918 | 701 | gb|AAU44084.1| putative MAP kinase phosphatase [*Oryza sativa*] | 1.00E-21 | | Up | MAC/TTG | |
| *11. Disease/defense* (18/255) | | | | | |  | | |
| S11_CY23_Contig3 | FG618672 | 331 | gb|AAW52718.1| peroxidase 4 [*Triticum monococcum*] | 2.00E-43 | | Up | MTC/TCA | |
| S11_CY23_Contig6 | FG618675 | 235 | gb|AAR25995.1| senescence-associated protein[*Pyrus communis*] | 5.00E-11 | | Down | MGT/TAC | |
| S11_CY23_Contig8 | FG618677 | 237 | dbj|BAE07207.1| salt-stress induced hydrophobic peptide [*Lophopyrum elongatum*] | 7.00E-23 | | Up | MAC/TTG | |
| S11_CY23_Contig31 | FG618700 | 212 | emb|CAJ83813.1| CHK1 checkpoint homolog [*Xenopus tropicalis*] | 2.00E-08 | | Up | MAC/TGA | |
| S11_CY23_17-3c | FG618827 | 214 | emb|CAJ83813.1| CHK1 checkpoint homolog [*Xenopus* *tropicalis*] | 2.00E-08 | | Up | MTC/TCA | |
| S11_CY23_Contig32* | FG618701 | 206 | gb|AAW52719.1| peroxidase 5 [*Triticum monococcum*] | 5.00E-27 | | Up | MTC/TAC | |
| S11_CY23_Contig46 | FG618715 | 304 | ref|XP_463067.1| peroxisomal membrane protein[*Oryza* *sativa*] | 4.00E-36 | | Up | MGT/TAC | |
| S11_CY23_396-1 | FG618891 | 411 | gb|AAW52718.1| peroxidase 4 [*Triticum monococcum*] | 6.00E-44 | | Up | MTG/TAG | |
| S11_CY23_Contig103 | FG618772 | 262 | ref|XP_550224.1| verticillium wilt disease resistance protein -like [*Oryza sativa*] | 7.00E-16 | | Up | MTG/TAG | |
| S11_CY23_Contig80 | FG618749 | 511 | gb|AAW78582.1| quinone reductase 2 [*Triticum* *monococcum*] | 2.00E-18 | | Up | MTC/TCA | |
| S11_CY23_Contig124 | FG618792 | 444 | gb|AAW78582.1| quinone reductase 2 [*Triticum* *monococcum*] | 1.00E-08 | | Up | MAC/TGA | |
| S11_CY23_Contig137 | FG618805 | 305 | gb|AAW78582.1| quinone reductase 2 [*Triticum* *monococcum*] | 9.00E-21 | | Down | MGT/TAC | |
| S11_CY23_Contig69* | FG618738 | 327 | gb|ABA94506.1| Glutathione S-transferase, [*Arabidopsis*] | 1.00E-31 | | Up | MTC/TCA | |
| S11_CY23_Contig91 | FG618760 | 406 | gb|AAA58585.2| hemolysin [*Acanthamoeba polyphaga*] | 6.00E-18 | | Up | MAC/TGA | |
| S11_CY23_Contig112 | FG618781 | 189 | gb|AAK60568.1| thaumatin-like protein [*Triticum* *aestivum*] | 7.00E-07 | | Up | MTG/TAG | |
| S11_CY23_397p-3* | FG618892 | 118 | gb|ABG22067.1| Leucine Rich Repeat family protein [*Oryza sativa*] | 2.00E-09 | | Up | MTG/TAG | |
| S11_CY23_181-4* | FG618829 | 159 | ref|XP_472189.1| OSJNBb0006N15.4 [*Oryza sativa*] | 6.00E-06 | | Up | MTC/TAC | |
| S11_CY23_231-1-4* | FG618855 | 224 | ref|XP_493854.1| pseudo-response regulator [*Oryza* *sativa*] | 5.00E-08 | | Down | MTG/TTG | |
| *12. Unclassified* (107/255) | | | | | |  | | |
| S11_CY23_Contig5* | FG618674 | 148 | No hits found |  | | Down | MGT/TAC | |
| S11_CY23_Contig7* | FG618676 | 169 | No hits found | - | | Up | MAC/TTG | |
| S11_CY23_Contig9 | FG618678 | 277 | No hits found | - | | Up | MTC/TAC | |
| S11_CY23_Contig13 | FG618682 | 290 | No hits found | - | | Up | MAC/TTG | |
| S11_CY23_Contig15* | FG618684 | 362 | No hits found | - | | Down | MAC/TTG | |
| S11_CY23_Contig21* | FG618690 | 196 | No hits found | - | | Down | MCA/TGT | |
| S11_CY23_Contig23 | FG618692 | 368 | No hits found | - | | Up | MAC/TGA | |
| S11_CY23_Contig36 | FG618705 | 236 | No hits found | - | | Up | MTC/TCA | |
| S11_CY23_Contig37 | FG618706 | 294 | No hits found | - | | Up | MAC/TGA | |
| S11_CY23_Contig38 | FG618707 | 296 | No hits found | - | | Down | MTC/TAC | |
| S11_CY23_Contig40 | FG618709 | 266 | No hits found | - | | Down | MTC/TTC | |
| S11_CY23_Contig42 | FG618711 | 309 | No hits found | - | | Down | MTC/TAC | |
| S11_CY23_Contig43* | FG618712 | 283 | No hits found | - | | Up | MCA/TGT | |
| S11_CY23_Contig45 | FG618714 | 251 | No hits found | - | | Down | MTG/TTG | |
| S11_CY23_Contig47 | FG618716 | 305 | No hits found | - | | Down | MGT/TAC | |
| S11_CY23_Contig48 | FG618717 | 242 | No hits found | - | | Up | MTC/TCA | |
| S11_CY23_Contig49 | FG618718 | 261 | No hits found | - | | Up | MTC/TAC | |
| S11_CY23_Contig53 | FG618722 | 254 | No hits found | - | | Up | MTC/TCA | |
| S11_CY23_Contig54 | FG618723 | 188 | No hits found | - | | Up | MCT/TTC | |
| S11_CY23_Contig56 | FG618725 | 169 | No hits found | - | | Up | MTG/TTG | |
| S11_CY23_Contig57* | FG618726 | 180 | No hits found | - | | Up | MCA/TCT | |
| S11_CY23_Contig58* | FG618727 | 201 | No hits found | - | | Up | MTG/TTG | |
| S11_CY23_Contig59* | FG618728 | 198 | No hits found | - | | Up | MTG/TTG | |
| S11_CY23_Contig60 | FG618729 | 133 | No hits found | - | | Down | MGA/TCT | |
| S11_CY23_Contig64 | FG618733 | 239 | No hits found | - | | Down | MTG/TAG | |
| S11_CY23_Contig73* | FG618742 | 359 | No hits found | - | | Up | MTG/TAG | |
| S11_CY23_Contig84 | FG618753 | 267 | No hits found | - | | Up | MAC/TGA | |
| S11_CY23_Contig85 | FG618754 | 171 | No hits found | - | | Down | MTC/TCA | |
| S11_CY23_Contig87 | FG618756 | 191 | No hits found | - | | Up | MAC/TGA | |
| S11_CY23_Contig92 | FG618761 | 171 | No hits found | - | | Up | MTC/TCA | |
| S11_CY23_Contig93* | FG618762 | 201 | No hits found | - | | Up | MTG/TAG | |
| S11_CY23_Contig98* | FG618767 | 237 | No hits found | - | | Up | MTG/TAG | |
| S11_CY23_Contig100* | FG618769 | 239 | No hits found | - | | Up | MGT/TAC | |
| S11_CY23_Contig101 | FG618770 | 251 | No hits found | - | | Down | MTC/TAC | |
| S11_CY23_Contig105* | FG618774 | 282 | No hits found | - | | Up | MGA/TCT | |
| S11_CY23_Contig108 | FG618777 | 319 | No hits found | - | | Down | MTG/TAG | |
| S11_CY23_Contig109 | FG618778 | 281 | No hits found | - | | Down | MTC/TAC | |
| S11_CY23_Contig115 | FG618784 | 403 | No hits found | - | | Up | MGA/TCT | |
| S11_CY23_Contig118* | FG618787 | 274 | No hits found | - | | Down | MAC/TGA | |
| S11_CY23_Contig119 | FG618788 | 194 | No hits found | - | | Up | MTC/TCA | |
| S11_CY23_Contig120 | FG618789 | 204 | No hits found | - | | Down | MGT/TAC | |
| S11_CY23_Contig123 | FG618791 | 271 | No hits found | - | | Up | MAC/TGA | |
| S11_CY23_Contig126* | FG618794 | 227 | No hits found | - | | Down | MAC/TGA | |
| S11_CY23_Contig127 | FG618795 | 319 | No hits found | - | | Up | MTC/TCA | |
| S11_CY23_Contig129 | FG618797 | 141 | No hits found | - | | Down | MTG/TTG | |
| S11_CY23_Contig130* | FG618798 | 283 | No hits found | - | | Down | MAC/TGA | |
| S11_CY23_Contig132 | FG618800 | 202 | No hits found | - | | Down | MGA/TCT | |
| S11_CY23_Contig134* | FG618802 | 163 | No hits found | - | | Up | MAC/TGA | |
| S11_CY23_Contig135 | FG618803 | 177 | No hits found | - | | Down | MTC/TCA | |
| S11_CY23_Contig138 | FG618806 | 159 | No hits found | - | | Up | MAC/TGA | |
| S11_CY23_Contig139 | FG618807 | 194 | No hits found | - | | Up | MGT/TAC | |
| S11_CY23_Contig140* | FG618808 | 173 | No hits found | - | | Down | MGT/TAC | |
| S11_CY23_Contig141* | FG618809 | 178 | No hits found | - | | Up | MTG/TTG | |
| S11_CY23_12-1-1b | FG618810 | 137 | No hits found | - | | Up | MGT/TAC | |
| S11_CY23_12-1-3* | FG618811 | 191 | No hits found | - | | Up | MAC/TGA | |
| S11_CY23_120-5 | FG618812 | 116 | No hits found | - | | Up | MAC/TGA | |
| S11_CY23_128-1* | GR955277 | 260 | No hits found | - | | Up | MGA/TCT | |
| S11_CY23_128-2* | FG618814 | 130 | No hits found | - | | Up | MCT/TTC | |
| S11_CY23_14-1-2c | FG618820 | 222 | No hits found | - | | Up | MAC/TTG | |
| S11_CY23_145-4 | FG618821 | 243 | No hits found | - | | Down | MTC/TCA | |
| S11_CY23_16-2c | FG618826 | 230 | No hits found | - | | Up | MGA/TCT | |
| S11_CY23_181-3* | FG618828 | 177 | No hits found | - | | Up | MTC/TCA | |
| S11_CY23_181-5* | FG618830 | 176 | No hits found | - | | Up | MTC/TAC | |
| S11_CY23_184-2 | FG618831 | 210 | No hits found | - | | Up | MCT/TTC | |
| S11_CY23_2-2c | FG618838 | 121 | No hits found | - | | Down | MTC/TAC | |
| S11_CY23_20-2b | FG618840 | 307 | No hits found | - | | Up | MTC/TCA | |
| S11_CY23_190s-5 | FG618833 | 246 | No hits found | - | | Down | MTC/TAC | |
| S11_CY23_193-4* | FG618834 | 297 | No hits found | - | | Up | MTC/TAC | |
| S11_CY23_205-1-2 | FG618842 | 177 | No hits found | - | | Up | MCT/TTC | |
| S11_CY23_205-1-3 | FG618843 | 163 | No hits found | - | | Down | MCT/TTC | |
| S11_CY23_209-5* | FG618846 | 117 | No hits found | - | | Down | MTC/TAC | |
| S11_CY23_213-1-3 | FG618848 | 187 | No hits found | - | | Up | MTC/TAC | |
| S11_CY23_216-1 | FG618851 | 123 | No hits found | - | | Up | MTC/TAC | |
| S11_CY23_22-2-2b* | FG618852 | 119 | No hits found | - | | Up | MGT/TAC | |
| S11_CY23_236-1* | FG618858 | 339 | No hits found | - | | Up | MTG/TTG | |
| S11_CY23_253s-4 | FG618864 | 121 | No hits found | - | | Up | MCA/TGT | |
| S11_CY23_257-1-1 | FG618865 | 173 | No hits found | - | | Up | MTG/TTG | |
| S11_CY23_257-1-3* | FG618866 | 169 | No hits found | - | | Up | MCA/TGT | |
| S11_CY23_257-1-5* | FG618868 | 180 | No hits found | - | | Up | MCA/TGT | |
| S11_CY23_257-3* | FG618869 | 172 | No hits found | - | | Up | MTG/TTG | |
| S11_CY23_25c-2 | FG618870 | 357 | No hits found | - | | Down | MTC/TCA | |
| S11_CY23_3-3b | FG618871 | 170 | No hits found | - | | Down | MGT/TAC | |
| S11_CY23_31c-4* | FG618872 | 124 | No hits found | - | | Up | MTC/TCA | |
| S11_CY23_344-1-3 | FG618873 | 141 | No hits found | - | | Up | MTG/TAG | |
| S11_CY23_346-1-5* | FG618874 | 157 | No hits found | - | | Up | MTG/TAG | |
| S11_CY23_35-4* | FG618877 | 160 | No hits found | - | | Down | MAC/TGA | |
| S11_CY23_352-5 | FG618878 | 192 | No hits found | - | | Down | MCA/TGT | |
| S11_CY23_354-3 | FG618879 | 209 | No hits found | - | | Up | MTG/TAG | |
| S11_CY23_376-4* | FG618884 | 226 | No hits found | - | | Up | MCA/TGT | |
| S11_CY23_39-1* | FG618889 | 180 | No hits found | - | | Up | MAC/TGA | |
| S11_CY23_394-3 | FG618890 | 170 | No hits found | - | | Up | MTG/TAG | |
| S11_CY23_42b-3* | FG618896 | 459 | No hits found | - | | Up | MGT/TAC | |
| S11_CY23_44-1-2b | FG618897 | 242 | No hits found | - | | Up | MGT/TAC | |
| S11_CY23_45-2* | FG618899 | 266 | No hits found | - | | Up | MAC/TGA | |
| S11_CY23_45-3* | GR955280 | 405 | No hits found | - | | Up | MCA/TGT | |
| S11_CY23_45s-3 | FG618900 | 147 | No hits found | - | | Up | MCA/TGT | |
| S11_CY23_45s-4* | FG618901 | 200 | No hits found | - | | Up | MAC/TGA | |
| S11_CY23_45x-1* | FG618902 | 210 | No hits found | - | | Up | MCA/TGT | |
| S11_CY23_48-5 | FG618904 | 231 | No hits found | - | | Down | MAC/TGA | |
| S11_CY23_50-2b | FG618905 | 173 | No hits found | - | | Down | MGT/TAC | |
| S11_CY23_51x-4 | FG618906 | 262 | No hits found | - | | Down | MAC/TGA | |
| S11_CY23_54-2b-3 | FG618907 | 254 | No hits found | - | | Up | MGT/TAC | |
| S11_CY23_54-2b-4 | FG618908 | 246 | No hits found | - | | Up | MGT/TAC | |
| S11_CY23_58-3 | FG618910 | 145 | No hits found | - | | Up | MCA/TGT | |
| S11_CY23_7-5c | FG618911 | 448 | No hits found | - | | Up | MTC/TCA | |
| S11_CY23_8b23s-1 | FG618915 | 160 | No hits found | - | | Down | MGT/TAC | |
| S11_CY23_8-1c* | FG618913 | 159 | No hits found | - | | Up | MTC/TCA | |
| *13. Unclear classification* (35/255) | | | | | |  | | |
| S11_CY23_Contig44 | FG618713 | 314 | gb|AAH53854.1| Unknown [*Homo sapiens*] | 8.00E-08 | | Down | MTC/TAC | |
| S11_CY23_Contig62* | FG618731 | 228 | dbj|BAD46368.1| unknown protein [*Oryza sativa*] | 1.00E-21 | | Down | MTG/TTG | |
| S11_CY23_Contig70 | FG618739 | 257 | gb|AAV25649.1| unknown protein [*Oryza sativa*] | 1.00E-35 | | Up | MCA/TGT | |
| S11_CY23_Contig71 | FG618740 | 259 | AAV25649.1| unknown protein [*Oryza sativa*] | 1.00E-35 | | Up | MCA/TGT | |
| S11_CY23_Contig86 | FG618755 | 331 | dbj|BAD46049.1| unknown protein [*Oryza sativa*] | 2.00E-07 | | Up | MTC/TCA | |
| S11_CY23_Contig97 | FG618766 | 356 | ref|XP_467147.1| unknown protein [*Oryza sativa*] | 3.00E-19 | | Up | MTG/TAG | |
| S11_CY23_Contig111 | FG618780 | 308 | gb|AAV25649.1| unknown protein [*Oryza sativa*] | 3.00E-36 | | Up | MTG/TTG | |
| S11_CY23_Contig10 | FG618679 | 310 | gb|AAT76998.1| hypothetical protein [*Oryza sativa*] | 1.00E-10 | | Up | MAC/TTG | |
| S11_CY23_Contig33 | FG618702 | 623 | dbj|BAE73006.1| hypothetical protein [*Macacafascicularis*] | 9.00E-10 | | Up | MTC/TAC | |
| S11_CY23_Contig34* | FG618703 | 220 | ref|XP_463926.1| hypothetical protein [*Oryza sativa*] | 3.00E-10 | | Down | MTG/TTG | |
| S11_CY23_Contig94 | FG618763 | 208 | gb|ABF97305.1| expressed protein [*Oryza sativa*] | 5.00E-14 | | Up | MTG/TAG | |
| S11_CY23_128-4 | FG618815 | 257 | gb|AAV25649.1| unknown protein [*Oryza sativa*] | 1.00E-35 | | Up | MAC/TTG | |
| S11_CY23_128-5 | FG618816 | 371 | gb|AAU44225.1| unknown protein [*Oryza sativa*] | 4.00E-09 | | Down | MAC/TTG | |
| S11_CY23_130-3* | FG618818 | 354 | ref|NP_565802.1|unknown protein [*Arabidopsis thaliana*] | 3.00E-09 | | Up | MAC/TTG | |
| S11_CY23_73-9 | FG618912 | 275 | ref|XP_482538.1| unknown protein [*Oryza sativa*] | 2.00E-19 | | Up | MAC/TGA | |
| S11_CY23_355-1 | FG618880 | 204 | ref|ZP_00569683.1| hypothetical protein[*Frankia*] | 6.00E-09 | | Up | MTG/TAG | |
| S11_CY23_148-3* | FG618823 | 308 | ref|NP_908682.1| P0426D06.23 [*Oryza sativa*] | 4.00E-24 | | Up | MAC/TTG | |
| S11_CY23_Contig2 | FG618671 | 206 | ref|ZP_00345902.1| hypothetical protein [*Nostoc* *punctiforme*] | 6.00E-15 | | Up | MTC/TCA | |
| S11_CY23_Contig12 | FG618681 | 368 | ref|ZP_00874766.1| hypothetical protein [*Streptococcus*] | 2.00E-30 | | Up | MAC/TTG | |
| S11_CY23_Contig24 | FG618693 | 259 | ref|ZP_00341848.1| hypothetical protein [*Lactobacillus*] | 6.00E-07 | | Down | MAC/TGA | |
| S11_CY23_Contig28 | FG618697 | 475 | gb|ABE87179.1| hypothetical protein [*Medicago* *truncatula*] | 2.00E-14 | | Up | MTC/TCA | |
| S11_CY23_Contig61 | FG618730 | 337 | ref|NP_781232.1| hypothetical protein [*Clostridium*] | 1.00E-18 | | Up | MTG/TAG | |
| S11_CY23_Contig74 | FG618743 | 324 | ref|ZP_00345902.1| hypothetical protein [*Nostoc* *punctiforme*] | 4.00E-22 | | Up | MTG/TTG | |
| S11_CY23_Contig79 | FG618748 | 246 | pir||F81737 hypothetical protein[*Chlamydia muridarum*] | 4.00E-10 | | Up | MCA/TGT | |
| S11_CY23_Contig113 | FG618782 | 444 | ref|YP_173415.1| hypothetical protein [*Nicotiana* *tabacum*] | 2.00E-13 | | Down | MAC/TTG | |
| S11_CY23_Contig117 | FG618786 | 478 | ref|ZP_00345902.1| hypothetical protein [*Nostoc* *punctiforme*] | 4.00E-22 | | Up | MTG/TAG | |
| S11_CY23_Contig128* | FG618796 | 606 | ref|XP_523482.1| hypothetical protein[*Pan troglodytes*] | 1.00E-06 | | Down | MAC/TGA | |
| S11_CY23_Contig131 | FG618799 | 300 | gb|ABE87179.1| hypothetical protein [*Medicago* *truncatula*] | 2.00E-22 | | Up | MTC/TCA | |
| S11_CY23_149-4 | FG618825 | 137 | gb|AAU03684.1| conserved hypothetical protein[*Rickettsia*] | 4.00E-11 | | Down | MAC/TTG | |
| S11_CY23_196-3* | FG618835 | 307 | ref|YP_052826.1| hypothetical protein OrniCp100 [*Oryzanivara*] | 2.00E-35 | | Down | MTC/TAC | |
| S11_CY23_216-1-3 | FG618850 | 160 | ref|YP_113253.1| hypothetical protein [*Methylococcus* *capsulatus*] | 1.00E-06 | | Up | MTC/TAC | |
| S11_CY23_226-1 | FG618853 | 181 | ref|YP_173415.1| hypothetical protein [*Nicotiana* *tabacum*] | 2.00E-19 | | Down | MTG/TTG | |
| S11_CY23_231-2* | GR955278 | 228 | gb|EEC68173.1| hypothetical protein [*Oryza sativa*] | 2.00-27 | | Up | MTG/TTG | |
| S11_CY23_355-5 | FG618881 | 207 | ref|NP_781232.1| hypothetical protein [*Clostridiu*m] | 8.00E-12 | | Up | MTG/TAG | |
| S11_CY23_361-1 | FG618885 | 233 | ref|ZP_00341848.1| hypothetical protein [*Lactobacillus* *gasseri*] | 8.00E-11 | | Up | MTG/TAG | |

^a^ The number of TDFs in each category and the total number of TDFs are indicated in the parentheses and separated by “/”. The TDFs marked with “*” were only found in the incompatible interaction when compared with the TDFs of the compatible interaction of the same wheat genotype, Suwon 11, with *Pst* pathotype CYR31 (Wang *et al*. 2009) using the BLASTN analysis; and those without “*” were common in the incompatible and compatible interactions.
