# Supplementary material for: Differential gene expression in incompatible interaction between wheat and stripe rust fungus revealed by cDNA-AFLP and comparison to compatible interaction
Source: BMC Plant Biol. 2010 Jan 12;10:9. doi: 10.1186/1471-2229-10-9 (PMC2817678; doi:10.1186/1471-2229-10-9)
Supplement: Additional file 2 — Selective primers used in cDNA-AFLP. displaying primers used for cDNA-AFLP. [file 1471-2229-10-9-S2.DOC]

| **Additional File 2.** Selective primers used in cDNA-AFLP | |
| --- | --- |
| *Mse*I primers | Sequences 5’-3’ |
| MAC | GATGAGTCCTGAGTAAAC |
| MAG | GATGAGTCCTGAGTAAAG |
| MCA | GATGAGTCCTGAGTAACA |
| MCT | GATGAGTCCTGAGTAACT |
| MGA | GATGAGTCCTGAGTAAGA |
| MGT | GATGAGTCCTGAGTAAGT |
| MTC | GATGAGTCCTGAGTAATC |
| MTG | GATGAGTCCTGAGTAATG |
| *Taq*I primers | Sequences 5’-3’ |
| TGA | GTAGACTGCGTACCGAGA |
| TGT | GTAGACTGCGTACCGAGT |
| TTC | GTAGACTGCGTACCGATC |
| TTG | GTAGACTGCGTACCGATG |
| TCT | GTAGACTGCGTACCGACT |
| TCA | GTAGACTGCGTACCGACA |
| TAG | GTAGACTGCGTACCGAAG |
| TAC | GTAGACTGCGTACCGAAC |
